# Rational Design of 3D Hierarchical Ternary SnO_2_/TiO_2_/BiVO_4_ Arrays Photoanode toward Efficient Photoelectrochemical Performance

**DOI:** 10.1002/advs.201902235

**Published:** 2019-12-09

**Authors:** Qin Pan, Aoshuang Li, Yuanlu Zhang, Yaping Yang, Chuanwei Cheng

**Affiliations:** ^1^ Shanghai Key Laboratory of Special Artificial Microstructure Materials and Technology School of Physics Science and Engineering Tongji University Shanghai 200092 P. R. China; ^2^ Institute of Dongguan‐Tongji University Dongguan Guangdong 523808 P. R. China

**Keywords:** arrays, composites, hierarchical structure, photoanodes, photoelectrochemical water spitting

## Abstract

BiVO_4_ as a promising semiconductor absorber is widely investigated as photoanode in photoelectrochemical water splitting. Herein, the rational design of 3D hierarchical ternary SnO_2_/TiO_2_/BiVO_4_ arrays is reported as photoanode for photoelectrochemical application, in which the SnO_2_ hierarchically hollow microspheres core/nanosheets shell arrays act as conductive skeletons, while the sandwiched TiO_2_ and surface BiVO_4_ are working as hole blocking layer and light absorber, respectively. Arising to the hierarchically ordered structure and synergistic effect between each component in the composite, the ternary SnO_2_/TiO_2_/BiVO_4_ photoanode enables high light harvesting efficiency as well as enhanced charge transport and separation efficiency, yielding a maximum photocurrent density of ≈5.03 mA cm^−2^ for sulfite oxidation and ≈3.1 mA cm^−2^ for water oxidation, respectively, measured at 1.23 V versus reversible hydrogen electrode under simulated air mass (AM) 1.5 solar light illumination. The results reveal that electrode design and interface engineering play important roles on the overall PEC performance.

## Graphical abstract

A novel 3D hierarchical ternary SnO_2_/TiO_2_/BiVO_4_ arrays photoanode is designed with excellent photoelectrochemical performance.

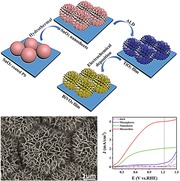

## 1 Introduction

Photoelectrochemical (PEC) water splitting that converts the solar light into hydrogen represents a green and sustainable way to address the energy crisis and environmental pollution issues simultaneously.^1^, ^2^, ^3^, ^4^ The overall PEC conversion efficiency is determined by light harvesting efficiency, charge separation efficiency, and surface reaction efficiency. As such, development of a suitable and efficient photoanode with high light absorption and charge separation efficiency is a key step to boost the PEC performance. In the past decades, metal oxides such as TiO_2_,^5^, ^6^ Fe_2_O_3_,^7^, ^8^, ^9^ and BiVO_4_ 10, 11, 12 have been widely investigated as photoanodes for PEC water splitting due to the characteristics of excellent photoactivity, low cost, and good stability. Among them, BiVO_4_ semiconductor is particularly attractive because it has a narrow band gap of ≈2.4 eV, suitable conduction band (0 V vs reversible hydrogen electrode, RHE) and valence band edges, high water splitting efficiency, and good chemical stability.^13^, ^14^, ^15^, ^16^, ^17^, ^18^ Nevertheless, the poor charge transport ability, short carrier diffusion lengths (≈70 nm), and high charge recombination rates of BiVO_4_ greatly limit its practical PEC performance. To address these problems, various strategies such as morphology design, element doping, host–guest heterojunctions and surface modifications are developed to enhance the performance.^19^, ^20^, ^21^, ^22^, ^23^, ^24^ Nanostructured electrode design is one effective route to enhance the light absorption and charge transport ability. For instance, 1D nanorods,^25^ 2D nanosheets,^26^ 3D inverse opals,^27^ 3D hierarchical structures,^28^, ^29^, ^30^ and 3D brochosomes‐like arrays^31^ have been reported to enhance the light harvesting and charge transport performance. Besides, the host–guest heterojunction electrode using two or more dissimilar materials to take the task of charge transport and light absorption, respectively, is another popular strategy to promote the charge transport and suppress the recombination of electron–hole pairs.^32^ For instance, BiVO_4_ as a light absorber has been combined with various other semiconductors to form a type II configuration such as TiO_2_/BiVO_4_,^33^, ^34^, ^35^, ^36^, ^37^, ^38^ WO_3_/BiVO_4_,^39^, ^40^, ^41^, ^42^ SnO_2_/BiVO_4_,^43^, ^44^ and Fe_2_O_3_/BiVO_4_.^45^ Among them, TiO_2_ is very attractive due to its relatively negative flat band potential and good chemical stability. Nevertheless, the intrinsically low mobility of TiO_2_ still greatly limits the overall performance of TiO_2_/BiVO_4_ heterojunction photoanode.

Herein, we report the rational design and fabrication of 3D hierarchical ternary SnO_2_/TiO_2_/BiVO_4_ arrays as photoanode for photoelectrochemical water splitting by combining multiple routes of colloid microspheres template, hydrothermal, atomic layer deposition (ALD), and electrodeposition. In this electrode design, the hierarchically hollow SnO_2_ microspheres@nanosheets arrays act as skeletons to support the TiO_2_ and BiVO_4_ layer ensuring fast and direct charge transport, the medium ALD conformal TiO_2_ layer works as a hole blocking layer to form a type II heterojunction with BiVO_4_, which is beneficial to reduce the charge recombination and improve the charge separation efficiency. Moreover, the 3D hierarchically ordered structures with large specific area and rich voids can further enhance the light harvesting and charge transport. As such, the hierarchical ternary SnO_2_/TiO_2_/BiVO_4_ arrays photoanode yields excellent PEC performance with a maximum photocurrent density of ≈5.03 mA cm^−2^ at 1.23 V versus RHE under air mass 1.5 light illumination, which is much superior to the counterparts of SnO_2_/TiO_2_/BiVO_4_ microspheres arrays and nanosheets arrays electrodes.

## 2 Results and Discussion

**Figure 1:**
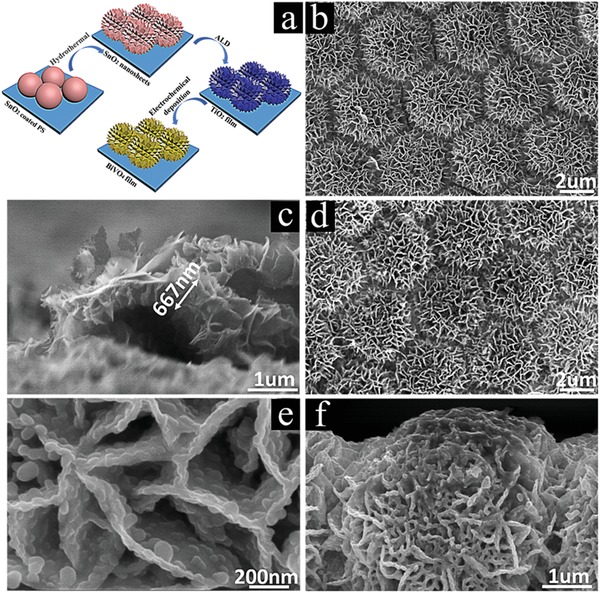
a) Scheme of the preparation processes of SnO_2_/TiO_2_/BiVO_4_ H‐NSs@HMs arrays. SEM images of SnO_2_ H‐NSs@HMs arrays: b) top view and c) cross‐section view. SnO_2_/TiO_2_/BiVO_4_ H‐NSs@HMs arrays: d,e) top view and f) cross‐section view.

The 3D SnO_2_/TiO_2_/BiVO_4_ hierarchical nanosheets@hollow microspheres (H‐NSs@HMs) arrays are fabricated by a combination of colloid spheres template, atomic layer deposition, hydrothermal growth, and electro deposition routes, as schematically illustrated in **Figure** **1**a. First, periodically ordered SnO_2_ hollow microsphere arrays were prepared on F‐doped SnO_2_ (FTO) substrate by colloid microsphere templates, ALD, and calcination processes. Noting that a thin ZnO layer was predeposited to protect the hexagonal closed‐packed polymer microspheres before the SnO_2_ deposition, as shown in Figure S1 in the Supporting Information. Then SnO_2_ nanosheets were prepared on the surfaces of each SnO_2_ microsphere by hydrothermal growth of SnS_2_ nanosheets and a subsequent thermal treatment in air to form hierarchical SnO_2_ hollow spheres core/nanosheets shell arrays (SnO_2_ H‐NSs/HMs). After that, one thin TiO_2_ layer was conformally deposited on the SnO_2_ surface by ALD. Finally, BiVO_4_ was deposited by an electrodeposition route, resulting in 3D SnO_2_/TiO_2_/BiVO_4_ H‐NSs@HMs.

Figure 1b shows top view of the SnO_2_ H‐NSs@HMs arrays. It can be seen that a number of ultrathin SnO_2_ nanosheets are uniformly assembled on each hollow microsphere, forming a hierarchical hollow nanosheets@microspheres core–shell structure with diameters of ≈4.5 µm. Noting that the hierarchically hollow microspheres are periodically distributed on the substrate, which would be beneficial to the light harvesting and charge transport. From the cross‐section view in Figure 1c, it can be observed that the thickness of individual nanosheet is ranging from 5 to 30 nm, and the whole nanosheets layer thickness is ≈667 nm. After the deposition of TiO_2_ and BiVO_4_, the ordered structure is still well preserved, as displayed in Figure 1d, indicating the conformal thin films deposition by ALD and electrodeposition, respectively. From a closer observation in Figure 1e,f, the nanosheet surfaces become more rough after the deposition of BiVO_4_ and the nanosheet thickness increased obviously. The BiVO_4_ layer thickness can be determined to be ≈20 nm. It is noteworthy that the BiVO_4_ films are consisted of lots of large grains with diameters from 30 to 50 nm, which are advantageous for further improving the light absorption and increasing the specific surface area. The mapping results and energy dispersive X‐ray spectrum (EDX) in Figures S2 and S3 in the Supporting Information recorded from the SnO_2_/TiO_2_/BiVO_4_ H‐NSs@HMs prove the existence and uniform elements distributions of Sn, Ti, Bi, V, and O. For comparison, SnO_2_/TiO_2_/BiVO_4_ nanosheets arrays, SnO_2_/TiO_2_/BiVO_4_ hollow microspheres arrays, and SnO_2_/TiO_2_/BiVO_4_ hierarchical hollow nanosheets@microspheres arrays with different diameters are also prepared, the corresponding scanning electron microscopy (SEM) images are provided in Figures S4–S6 in the Supporting Information, respectively.

**Figure 2:**
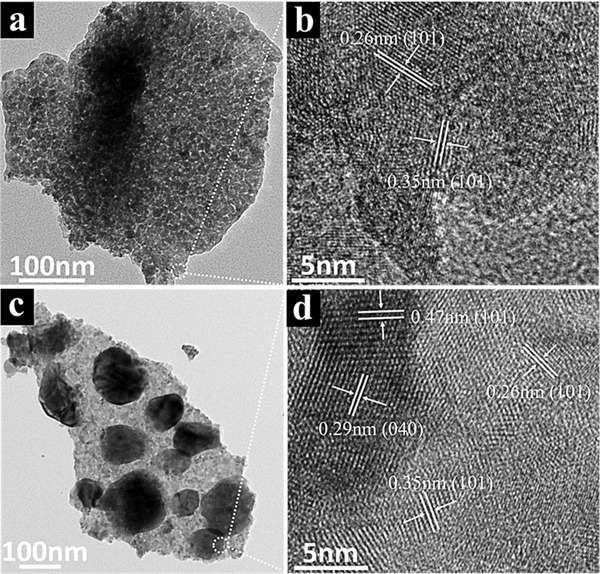
TEM image and high‐resolution TEM images of a,b) SnO_2_/TiO_2_ H‐NSs@HMs arrays and c,d) SnO_2_/TiO_2_/BiVO_4_ H‐NSs@HMs arrays.

The transmission electron microscopy (TEM) image of the SnO_2_/TiO_2_ nanosheet in **Figure** **2**a confirms that TiO_2_ are uniformly covered on the SnO_2_ nanosheet, suggesting the uniform and conformal film deposition by ALD. It can be seen that the nanosheet is consisted of nanoparticles with lots of mesopores. The mesoporous structures would be beneficial to increase the interface area and facilitate the electrolyte infiltration. The measured spacing of 0.26 and 0.35 nm in Figure 2b can be assigned to tetragonal phase of SnO_2_ and anatase phase of TiO_2_, respectively. After the BiVO_4_ deposition, a number of nanoplates are uniformly distributed on the SnO_2_/TiO_2_ nanosheet, as shown in Figure 2c, which is in agreement of the SEM observations. The lattice spacings of 0.47, 0.29, 0.26, and 0.35 nm in Figure 2d correspond to the (110), (040) planes of monoclinic BiVO_4_, (101) planes of tetragonal SnO_2_, and (101) planes of anatase TiO_2_, respectively.

**Figure 3:**
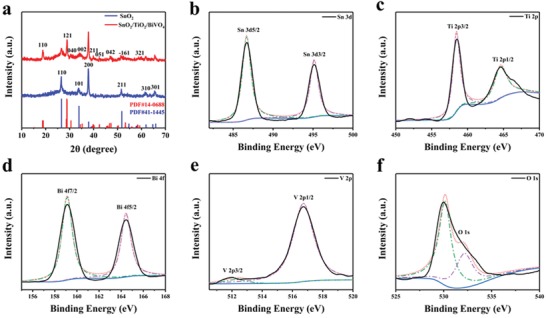
a) X‐ray diffraction (XRD) pattern of SnO_2_ and SnO_2_/TiO_2_/BiVO_4_ H‐NSs@HMs arrays. XPS core elemental spectrum recorded from SnO_2_/TiO_2_/BiVO_4_ H‐NSs@HMs: b) Sn 3d, c) Ti 2p, d) Bi 4f, e) V 2p, and f) O 1s.

The crystal structure of SnO_2_ H‐NSs@HMs and SnO_2_/TiO_2_/BiVO_4_ H‐NSs@HMs were analyzed by X‐ray diffraction (XRD). As displayed in **Figure** **3**a, the peaks of 26.41°, 34.06°, 37.88°, 51.67°, 61.87°, and 65.94° correspond to (110), (101), (200), (211), (310), and (301) planes of tetragonal phase of SnO_2_ (Joint Committee on Powder Diffraction Standards (JCPDS) No. 41‐1445), while the other peaks can be assigned to monoclinic BiVO_4_ phase (JCPDS No. 14‐0688). The chemical composition and surface states of SnO_2_/TiO_2_/BiVO_4_ H‐NSs@HMs was characterized by X‐ray photoelectron spectrum (XPS). The full survey spectrum in Figure S7 in the Supporting Information records the V, Sn, Ti, Bi, and O elements. The high‐resolution Sn element XPS spectra in Figure 3b show two peaks at 486.7 and 495.1 eV respectively, corresponding to Sn^4+^ in SnO_2_. The two satellite peaks of the high‐resolution Ti 2p_3/2_ and Ti 2p_1/2_ in Figure 3c centered at binding energies 458.5 and 464.6 eV are typical characteristic Ti^4+^ binding in TiO_2_. The signal at Ti 2p_1/2_ is asymmetric and has shoulders on the lower energy side, which might be related to the combination of BiVO_4_ and SnO_2_.^46^, ^47^, ^48^ The high‐resolution Bi, V, and O spectra can help identify the built‐in potential in the interface. The binding energy located at 159.1 and 164.5 eV in Figure 3d correspond to the Bi 4f_7/2_ orbit and Bi 4f_5/2_ orbit, respectively. The splitting peaks at 512.0 and 516.7 eV in Figure 3e correspond to V 2p_3/2_ orbit and V 2p_1/2_ orbit, respectively.^49^, ^50^, ^51^, ^52^ The high‐resolution O 1s XPS spectra in Figure 3f show two peaks, one has the binding energy at 530.0 eV is attributed to the lattice oxygen of the Sn—O, Ti—O, Bi—O, and V—O, and the other peak at 532.0 eV can be assigned to the hydroxide in O—H and adsorbed oxygen.^53^, ^54^ The high‐resolution Fe 2p spectra and Ni 2p spectra results in Figure S8 in the Supporting Information indicated that FeOOH/NiOOH catalyst were successfully deposited on the SnO_2_/TiO_2_/BiVO_4_ hierarchical structures.^24^

**Figure 4:**
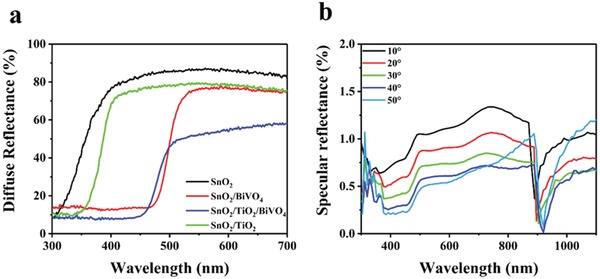
a) UV–vis diffuse reflectance spectra of SnO_2_, SnO_2_/TiO_2_, SnO_2_/BiVO_4_, SnO_2_/TiO_2_/BiVO_4_ H‐NSs@HMs arrays. b) Specular reflectance spectra of SnO_2_/TiO_2_/BiVO_4_ H‐NSs@HMs arrays at incident angles of 10°, 20°, 30°, 40°, and 50°.

The light absorption characteristics of as fabricated samples are characterized by diffuse reflectance spectrum and transmittance spectrum. **Figure** **4**a shows the diffuse reflectance spectrum of SnO_2_, SnO_2_/TiO_2_, SnO_2_/TiO_2_/BiVO_4_, and SnO_2_/BiVO_4_ H‐NSs@HMs arrays. For the SnO_2_ and SnO_2_/TiO_2_ H‐NSs@HMs arrays, the observed absorption edges of ≈310 and ≈350 nm are corresponding to the bandgap of SnO_2_ and TiO_2_, respectively. After the deposition of BiVO_4_, the absorption edge redshifts to ≈450 nm, which is in agreement of the bandgap absorption of BiVO_4_. It is worth noting that the SnO_2_/TiO_2_/BiVO_4_ H‐NSs@HMs arrays display the lowest light reflectance and light transmittance intensity in the measured wavelength of 300–700 nm among all the referenced samples, as shown in Figure 4a and Figure S9 in the Supporting Information, indicating that the SnO_2_/TiO_2_/BiVO_4_ H‐NSs@HMs arrays possess the highest light capturing ability, due to the ordered hierarchy structure as well as synergistic effect between each component in the composite. To demonstrate the omnidirectional light harvesting ability of the SnO_2_/TiO_2_/BiVO_4_ H‐NSs@HMs arrays, the specular reflection spectra at different light incidence angles are measured. As shown in Figure 4b, the SnO_2_/TiO_2_/BiVO_4_ H‐NSs@HMs arrays exhibits excellent omnidirectional antireflection characteristics with light reflection intensity below 1% in a wide wavelength range of 300 to 1100 nm. The excellent omnidirectional light harvesting ability could be due to the periodically ordered hierarchy structures that result in multiple light scattering, increased optical paths, and reduced light reflectance.^29^, ^55^

**Figure 5:**
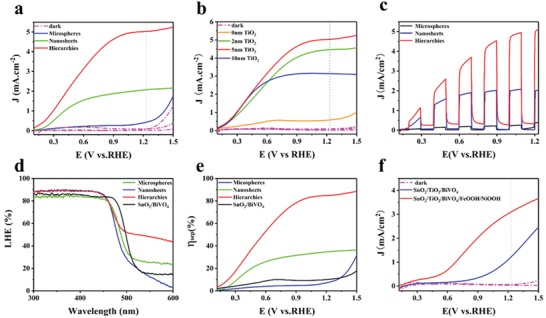
LSV curves measured under simulated light and dark conditions in 0.5 m Na_2_SO_4_ solution with 0.1 m Na_2_SO_3_ a) SnO_2_/TiO_2_/BiVO_4_ HMs, SnO_2_/TiO_2_/BiVO_4_ NSs, and SnO_2_/TiO_2_/BiVO_4_ H‐NSs@HMs arrays. b) Comparison of SnO_2_/TiO_2_/BiVO_4_ H‐NSs@HMs arrays with different TiO_2_ layer thickness. c) Chopped photocurrent‐potential curves of SnO_2_/TiO_2_/BiVO_4_ HMs, SnO_2_/TiO_2_/BiVO_4_ NSs, and SnO_2_/TiO_2_/BiVO_4_ H‐NSs@HMs arrays. d,e) Light harvesting efficiency (LHE) and charge separation efficiency (η_sep_) of SnO_2_/TiO_2_/BiVO_4_ HMs, SnO_2_/TiO_2_/BiVO_4_ NSs, SnO_2_/BiVO_4_ H‐NSs@HMs arrays, and SnO_2_/TiO_2_/BiVO_4_ H‐NSs@HMs arrays. f) PEC water splitting performance of SnO_2_/TiO_2_/BiVO_4_ H‐NSs@HMs arrays with and without FeOOH/NiOOH water oxidation catalyst.

The PEC performance of as‐fabricated samples is evaluated by linear sweep voltammetric (LSV) measurement under AM1.5 simulated light illumination (100 mW cm^−2^). The measurement was conducted in 0.5 m Na_2_SO_4_ electrolyte with 0.1 m Na_2_SO_3_ as a hole scavenger due to the fast kinetics of the sulfite oxidation. **Figure** **5**a shows the comparison LSV curves of three electrodes with different morphology measured in the dark and light conditions of SnO_2_/TiO_2_/BiVO_4_ hollow microspheres (HMs) arrays, SnO_2_/TiO_2_/BiVO_4_ nanosheets (NSs) arrays, and SnO_2_/TiO_2_/BiVO_4_ H‐NSs@HMs arrays. It can be seen that all the electrodes exhibit negligible photocurrent density under dark condition. While under light illumination, obvious photocurrent density is observed. The SnO_2_/TiO_2_/BiVO_4_ H‐NSs@HMs arrays photoanode shows the highest PEC performance with a maximum photocurrent density of 5.03 mA.cm^−2^ at 1.23 V versus RHE, which is much higher than that of SnO_2_/TiO_2_/BiVO_4_ hollow microspheres arrays and SnO_2_/TiO_2_/BiVO_4_ nanosheets arrays. Our results are superior to most of the recent reports on BiVO_4_ based photoanodes, as listed in Table S1 in the Supporting Information. The significantly improved PEC performance could be due to the hierarchically ordered structure that provides large specific surface area and efficient interface contact, resulting in improved light absorption and charge separation efficiency. To further illustrate the important role of TiO_2_ layer on the PEC performance, SnO_2_/TiO_2_/BiVO_4_ H‐NSs@HMs samples with different TiO_2_ layer thickness were prepared. As shown in Figure 5b, all the SnO_2_/TiO_2_/BiVO_4_ H‐NSs@HMs arrays photoanodes exhibit much better PEC performance in contrast to that of SnO_2_/BiVO_4_ H‐NSs@HMs arrays. The greatly improved performance might be due to the important role of TiO_2_ acting as a hole blocking layer to improve the charge separation efficiency and reduce the recombination rates. Noting that the optimized TiO_2_ layer thickness is ≈5 nm because a thicker TiO_2_ layer would affect the charge transport and reduce the PEC performance. Moreover, the effect of microspheres sizes and BiVO_4_ layer thickness on the PEC performance of SnO_2_/TiO_2_/BiVO_4_ H‐NSs@HMs arrays photoanode was also investigated. As shown in Figure S10 in the Supporting Information, the SnO_2_/TiO_2_/BiVO_4_ H‐NSs@HMs arrays electrode with diameters of ≈4.5 µm and moderate BiVO_4_ layer thickness display the optimized performance. Figure 5c shows the LSV curves measured under light on/off cycles, all the photoanodes show fast photoresponse and recovery time. The photoelectrochemical sulfite oxidation efficiency can be expressed as Equation (1)

(1) $$J_{\mathrm{sulfite}}=J_{\max}\times \eta_{\mathrm{abs}}\times \eta_{\mathrm{sep}}\times \eta_{\mathrm{trans}}$$

where *J*_sulftie_ is defined as the photocurrent density of sulfite oxidation, *J*_max_ is defined the maximum photocurrent density, η_abs_ is the light absorption efficiency, η_sep_ is the charge separation efficiency, and η_tran_ *_s_* is the charge transfer efficiency. Assuming that η_trans_ is *≈*100% at 1.23 V versus RHE in the presence of hole scavengers, the η_abs_ × η_sep_  = *J*_sulftie_/*J*_max_. In our system, the *J*_max_ is calculated to be ≈6.67 mA.cm^−2^ by using photon energy and solar photon flux at 300–515 nm,^14^, ^56^ the calculation details are provided in the Supporting information. The η_abs_ is determined by the light harvesting efficiency (LHE), i.e., LHE (%) = 100% − *R*(λ) (%) − *T*(λ) (%), as plotted in Figure 5d. Hence, the η_sep_ can be determined. As shown in Figure 5e, the SnO_2_/TiO_2_/BiVO_4_ H‐NSs@HMs arrays electrode shows the highest charge separation efficiency of 84.9% in contrast to that of SnO_2_/BiVO_4_ H‐NSs@HMs arrays, SnO_2_/TiO_2_/BiVO_4_ HMs arrays and SnO_2_/TiO_2_/BiVO_4_ NSs arrays. Moreover, the PEC water splitting performance SnO_2_/TiO_2_/BiVO_4_ H‐NSs@HMs arrays with and without FeOOH/NiOOH catalyst were measured in 0.5 m Na_2_SO_4_ electrolyte. As shown in Figure 5f, the FeOOH/NiOOH‐modified SnO_2_/TiO_2_/BiVO_4_ H‐NSs@HMs arrays photoanode shows significantly enhanced performance, yielding a maximum photocurrent density value of ≈3.1 mA cm^−2^ at 1.23 V versus RHE. This might be due to the role of the FeOOH and NiOOH dual water oxidation catalyst, which could facilitate the hole transfer efficiency and improve the water oxidation kinetics.

**Figure 6:**
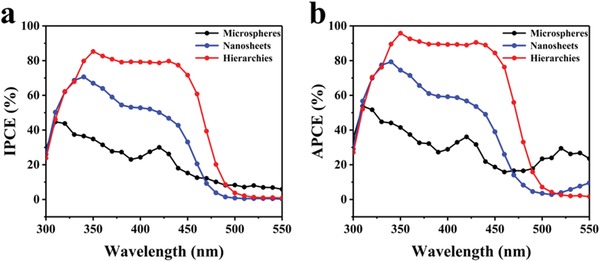
a) IPCE spectra and b) spectra APCE of SnO_2_/TiO_2_/BiVO_4_ hollow microspheres arrays, SnO_2_/TiO_2_/BiVO_4_ nanosheets arrays and SnO_2_/TiO_2_/BiVO_4_ H‐NSs@HMs arrays collected at 1.23 V versus RHE.

In order to study the wavelength‐dependent photoactivity of as‐fabricated photoanodes, the incident photoelectric conversion efficiency (IPCE) tests were conducted in a three‐electrode system at 1.23 V versus RHE. The IPCE can be calculated by the following formula (2)

(2) $$\mathrm{IPCE}=\frac{1024I}{\lambda J_{\mathrm{light}}}$$

where *I* is the photocurrent density, λ is the wavelength of the incident light, and *J*_light_ represents the incident light power intensity. As displayed in **Figure** **6**a, the SnO_2_/TiO_2_/BiVO_4_ H‐NSs@HMs arrays electrode displays the highest IPCE values in the wavelength of 325–475 nm in contrast to that of the other two reference samples and a maximum IPCE value of ≈85% was reached at 350 nm. The improved IPCE results of SnO_2_/TiO_2_/BiVO_4_ H‐NSs@HMs arrays could be due to its high light absorption and charge separation efficiency.^57^, ^58^, ^59^ To exclude the light absorption effect in the three different electrodes, the absorption photoelectric conversion efficiency (APCE) is used to determine quantum efficiency based on the IPCE and UV–vis absorption spectra results.^31^ As shown in Figure 6b, the SnO_2_/TiO_2_/BiVO_4_ H‐NSs@HMs also yield the highest APCE efficiency in the wavelength range of 300–500 nm. This would be attributed to the ordered structure that provides large interface contact area, facilitating the charge transport, separation, and collection process.

**Figure 7:**
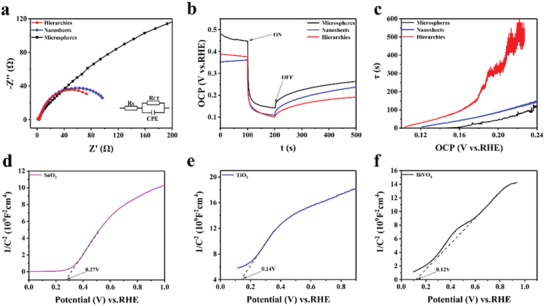
a) EIS spectra of SnO_2_/TiO_2_/BiVO_4_ hollow microspheres arrays, SnO_2_/TiO_2_/BiVO_4_ nanosheets arrays, and SnO_2_/TiO_2_/BiVO_4_ H‐NSs@HMs arrays measured under open circuit with light illumination. b,c) Open circuit voltage decay (OCVD) and electron lifetime of SnO_2_/TiO_2_/BiVO_4_ HMs, SnO_2_/TiO_2_/BiVO_4_ NSs, and SnO_2_/TiO_2_/BiVO_4_ H‐NSs@HMs arrays. Mott–Schottky plots measured in the dark at frequency of 500 Hz: d) SnO_2_, e) planar TiO_2_ films, and f) BiVO_4_ films.

In order to understand the charge transfer process of SnO_2_/TiO_2_/BiVO_4_ electrodes, electrochemical impedance spectroscopy (EIS) measurements on the three different structures were conducted under open circuit with AM1.5 simulated light illumination. As shown in the EIS spectra in **Figure** **7**a, the semicircle radius in the middle frequency range represents the charge transfer resistance at the photoelectrode/electrolyte interface, the SnO_2_/TiO_2_/BiVO_4_ H‐NSs@HMs photoanode shows the lowest charge transfer resistance in comparison to the counterparts, suggesting that the hierarchically ordered electrode has improved charge transport and separation efficiency. Besides, the SnO_2_/TiO_2_/BiVO_4_ electrode shows lower charge transfer resistance in contrast to that of SnO_2_/TiO_2_ and SnO_2_/BiVO_4_ samples (as shown in Figure S12, Supporting Information), indicating that the TiO_2_ layer is beneficial to facilitate the charge transfer process.

The minority carrier lifetime (τ) is an important parameter affecting the overall conversion efficiency of semiconductor electrodes. The recombination kinetics of as‐fabricated SnO_2_/TiO_2_/BiVO_4_ samples were studied by open circuit voltage decay (OCVD) route, in which the photovoltage decay rate is directly related to the electron lifetime, and the lifetime of the injected electron can be obtained according to Equation (3), 60

(3) $$\tau_{n}=\frac{k_{B}T}{e}{\left(\frac{dV_{\mathrm{oc}}}{dt}\right)}^{-1}$$

where *k*_B_ is the Boltzmann constant, *T* is the temperature, *e* is the electron charge, *d*_Voc_ is the amount of change in photovoltage, and d*t* is the amount of change in time. The *k*_B_ *T/e* is ≈0.026 V at room temperature. Therefore, the lifetime can be determined based on the OCVD curves in Figure 7c. As shown in Figure 7d, the SnO_2_/TiO_2_/BiVO_4_ H‐NSs@HMs arrays photoanode show prolonged carrier life time in comparison with the counterparts of SnO_2_/TiO_2_/BiVO_4_ HMs and SnO_2_/TiO_2_/BiVO_4_ NSs, resulting in the reduced recombination rates and improved charge separation efficiency. Moreover, the SnO_2_/TiO_2_/BiVO_4_ H‐NSs@HMs also have increased carrier life time in comparison to that of SnO_2_/TiO_2_ and SnO_2_/BiVO_4_ H‐NSs@HMs arrays, further indicating that the medium TiO_2_ layer can facilitate the charge separation and reduce the charge recombination, leading to enhanced PEC performance, as shown in Figure S13 in the Supporting Information.

The flat band potential (*V*_fb_) is measured by the open circuit potential of the photocurrent. The flat band potential *V*_fb_ of SnO_2_, TiO_2_, and BiVO_4_ in the composite can be determined by the Mott–Schottky curve as Equation (4)

(4) $$1/c^{2}=\left(\frac{2}{e\varepsilon \varepsilon_{0}N_{d}}\right)\left(\frac{V_{a}-V_{\mathrm{fb}}-K_{B}T}{e}\right)$$

where *C* is the capacitance of the space charge layer, *e* is the electron charge, ε is the dielectric constant, ε_0_ is the dielectric constant of the vacuum, *N*_d_ is the density of the electron donor, *V*_a_ is the applied potential, *V*_fb_ is the flat potential, *K*_B_ is the Boltzmann constant, and *T* is temperature. The *V*_fb_ is determined by a linear fit of the *x* intercept as function of 1/*c^2^*. As shown in Figure 7d–f, the *V*_fb_ of SnO_2_, TiO_2_, and BiVO_4_ are determined to be 0.27, 0.14, and 0.12 V (vs RHE), respectively. Assuming that the gap between the conduction band bottom edge and *V*_fb_ can be ignored, the difference in the *V*_fb_ values confirms the feasible electron transfer from BiVO_4_ to TiO_2._

To further determine the valence band positions, ultraviolet photoelectron spectroscopy (UPS) measurements on SnO_2_, TiO_2,_ and BiVO_4_ were conducted. The valence band edges were calculated using the photon energy of He I (21.22 eV) and the low kinetic energy cutoff of the spectrum and the valence band maximum with respect to the Fermi level (*E*_F_).^61^ As shown in Figure S14a in the Supporting Information, the valence band positions of SnO_2_, TiO_2_, and BiVO_4_ are found to be 7.93, 7.58, and 6.93 eV versus the *E*_vac_ or 3.49, 3.14, and 2.49 eV on the RHE.

**Figure 8:**
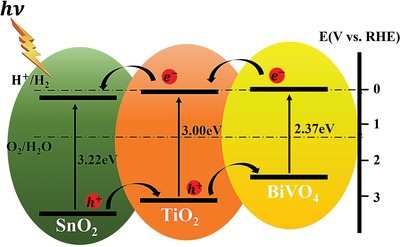
The electronic transfer mechanism of SnO_2_/TiO_2_/BiVO_4_ H‐NSs@HMs arrays.

The electronic transfer mechanism can be illustrated in **Figure** **8**. The band alignment was confirmed by the optical bandgap from the UV–vis absorption spectra (Figure S14b, Supporting Information) and the valence band edges measured by UPS. In the SnO_2_/TiO_2_/BiVO_4_ heterojunctions system, the TiO_2_/BiVO_4_ and TiO_2_/SnO_2_ build cascade structured band alignment, which significantly improve the charge separation and reduce the charge recombination at the interface.^61^, ^62^, ^63^ Under light excitation, both BiVO_4_ and TiO_2_ can absorb photons and generate electron–hole pairs. The photogenerated electrons in BiVO_4_ facilitated to transfer to the conduction band of TiO_2_ due to the type II configuration and then transported by the conductive SnO_2_ skeletons.^64^ The significantly enhanced PEC performance can be due to the following reasons. First, the hierarchically ordered hollow microspheres/nanosheets arrays structure can provide large specific area and rich porosity, as result in improved light harvesting efficiency. Second, the type II heterojunction and excellent contact between the TiO_2_ and BiVO_4_ facilitate the charge separation efficiency. Third, the enhanced charge injection and charge transfer process in the electrode/electrolyte interface of ordered SnO_2_/TiO_2_/BiVO_4_ also contribute the improved charge transport and separation. The last but not the least, the synergistic effect of each component in the ternary composite leads to the improved overall performance.

## 3 Conclusions

In summary, we have designed and fabricated a 3D hierarchical ternary SnO_2_/TiO_2_/BiVO_4_ arrays photoanode for photoelectrochemical application. The unique hierarchically ordered electrode design and synergistic effect between each component in the SnO_2_/TiO_2_/BiVO_4_ photoanode led to improved charge transport efficiency, light harvesting efficiency, and charge separation efficiency, as a result in significantly improved PEC performance. Our results demonstrate that electrode design and interface engineering play important roles to boost the overall PEC performance and would open new opportunities for design of various heterojunction photoelectrodes for solar energy conversion.

## 4 Experimental Section

*Preparation of SnO_2_/TiO_2_/BiVO_4_ Hierarchical Nanosheets@Hollow Microspheres (H‐NSs@HMs) Arrays*: The preparation processes SnO_2_/TiO_2_/BiVO_4_ hierarchical nanosheets@hollow microspheres (H‐NSs@HMs) arrays can be divided into three steps. First, SnO_2_ H‐NSs@HMs arrays on FTO substrate were prepared by colloid spheres template, ALD, and hydrothermal growth process. Second, TiO_2_ films with different thicknesses were deposited on SnO_2_ H‐NSs@HMs by ALD. The sample was placed in the ALD apparatus, and TiCl_4_ and H_2_O were used as Ti and O source, respectively. The temperature was 80 °C and the pressure was 11 × 10^3^ Pa. Finally, on layer of BiVO_4_ was deposited on the surface by electrochemical deposition route^35^ and the thickness was controlled by deposition times. For comparison, SnO_2_/TiO_2_/BiVO_4_ hollow microspheres array and SnO_2_/TiO_2_/BiVO_4_ nanosheets arrays are also prepared. The experiment details are described in the Supporting information.

*Characterizations*: The surface morphology of the samples was observed using field electron and ion company (FEI) Sirion 200 field emission SEM. The microstructure of the sample was characterized using JEM 2014F TEM. The phase analysis was carried out by XRD (D8‐Advance, Bruker Miller). The content of each component was determined by energy dispersive X‐ray Spectroscopy (EDX) of an X‐max 80 (Oxford) model. X‐ray photoelectron spectroscopy (Kratos Axis Ultra delay line detector (DLD)) characterizes elemental composition and valence state. Ultraviolet photoelectron spectroscopy measurements were conducted out on an ThermoFisher ESCALAB 250 XI spectrometer with a He discharge lamp (He I line, 21.22 eV) as the excitation source and a sample bias of −5 V was applied. The diffuse reflectance spectra were obtained on ultraviolet—see spectrophotometer (Hatachi, U3900H) and Specular reflectance spectra were recorded on Lambda 950 spectrophotometer.

*PEC Measurements*: The PEC performance of the samples was measured by electrochemical workstation (CS350, CS instrument) with simulated AM1.5G (100 mW cm^−2^) light illumination. The samples, Ag/AgCl (3M KCl), and Pt foil were employed as working electrodes, reference electrode, and counter electrode, respectively. The electrolyte was 0.5 m Na_2_SO_4_ mixed solution with 0.1 m Na_2_SO_3_ as hole scavenger. The measured potentials was converted from Ag/AgCl to the RHE by the following formula (5)

(5) $$E_{\mathrm{RHE}}=E_{\mathrm{Ag/AgCl}}+0.059\mathrm{pH}+0.197$$

*E*_RHE_ is the potential of the RHE, *E*_Ag/AgCl_ is the potential of the reference electrode Ag/AgCl, and pH is the pH value of the electrolyte. Simulated sunlight with 100 mW cm^−2^ was offered by a solar simulator (Zolix SS150). EIS spectra were measured in the frequency range of 10^−1^ to 10^5^ Hz under a 10 mV amplitude. IPCE values were obtained by IPCE system (Zolix Solar cell Scan100) in the wavelength range of 300–550 nm at the bias of 1.23 V versus RHE.

## Conflict of Interest

The authors declare no conflict of interest.

## Supporting information

Supporting Information
