# Supplementary material for: Rational Design of 3D Hierarchical Ternary SnO2/TiO2/BiVO4 Arrays Photoanode toward Efficient Photoelectrochemical Performance
Source: Adv Sci (Weinh). 2019 Dec 9;7(3):1902235. doi: 10.1002/advs.201902235 (PMC7001624; doi:10.1002/advs.201902235)
Supplement: Supplementary file 1 — Supporting Information [file ADVS-7-1902235-s001.pdf]

Copyright WILEY-VCH Verlag GmbH & Co. KGaA, 69469 Weinheim, Germany, 2019.

ADVANCED SCIENCE
Open Access

Supporting Information

for *Adv. Sci.,* DOI: 10.1002/advs.201902235

Rational Design of 3D Hierarchical Ternary $SnO_2/TiO_2/BiVO_4$ Arrays Photoanode toward Efficient Photoelectrochemical Performance

*Qin Pan, Aoshuang Li, Yuanlu Zhang, Yaping Yang, and Chuanwei Cheng**

Copyright WILEY-VCH Verlag GmbH & Co. KGaA, 69469 Weinheim, Germany, 2019.

Supporting Information

for *Adv Sci.*, DOI: 10.1002/advs. ((please add manuscript number))

Rational design of three-dimensional hierarchical ternary $SnO_2/TiO_2/BiVO_4$ arrays photoanode towards efficient photoelectrochemical performance

Qin Pan[1], Aoshuang Li[1], Yuanlu Zhang[1], Yaping Yang[1], Chuanwei Cheng[1, 2]*

[*]Ms Qian Pan, Ms Aoshuang Li, Ms Yuanlu Zhang, Prof. Yaping Yang and Prof. Chuanwei Cheng

Shanghai Key Laboratory of Special Artificial Microstructure Materials and Technology, School of Physics Science and Engineering, Tongji University, Shanghai 200092, P. R. China.
E-mail: cwcheng@tongji.edu.cn

[*] Prof. C. W. Cheng

Institute of Dongguan-Tongji University, Dongguan, Guangdong, 523808, P. R. China

Experimental details

Preparation of SnO_2 H-NSs@HMs arrays:

First, A single-layer microsphere was prepared on FTO substrate by a self-assembly method. Second, a ZnO thin film is deposited on the assembled hexagonal close-packed microspheres with a film thickness of 20 nm by ALD. Next, SnO_2 was deposited in the same manner with $SnCl_4$ and H_2O as the Sn source and the O source, and the thickness was 15 nm. The temperature is 80 °C and the pressure is 11×10^3 Pa. The pulse time of the source and the purge time of the nitrogen were 0.1 s and 18 s, respectively. The thickness of the film is precisely controlled by the number of ALD cycles. Then, the SnO_2 nanosheets were prepared by hydrothermal growth of SnS_2 and a subsequent calcination in air. The solution was mixed with crystalline $SnCl_4$ (0.03 M) and CH_3CSNH_2 (0.08 M) in ethanol. After stirring evenly, the solution was poured into a Poly tetra fluoroethylene (PTFE) lining, and the sample was placed upside down in a bottle and sealed for hydrothermal growth at a temperature of 80 °C for 1 h. Next, an air annealing treatment was performed at 450 °C for 2 hours, and the temperature rising rate was 3.75 °C/min, and the generated SnS_2 was converted into SnO_2.

Preparation of FeOOH/NiOOH catalyst:

The FeOOH/NiOOH catalyst was prepared by a photo-assisted electrochemical deposition route. Different amounts of FeOOH and NiOOH are deposited by controlling the amount of charge. An aqueous solution of $FeSO_4$ (0.1 M) was subjected to photo-assisted electrochemical deposition, and the amount of deposition was 120 mC at 0.25 V versus Ag/AgCl. The light source is the 150 W xenon lamp AM1.5G under simulated sunlight. An aqueous solution of Ni_2SO_4 (0.1 M) was adjusted to pH 7.0 with KOH (0.1 M), and the charge of 35 mC was deposited at 0.11 V versus Ag/AgCl.

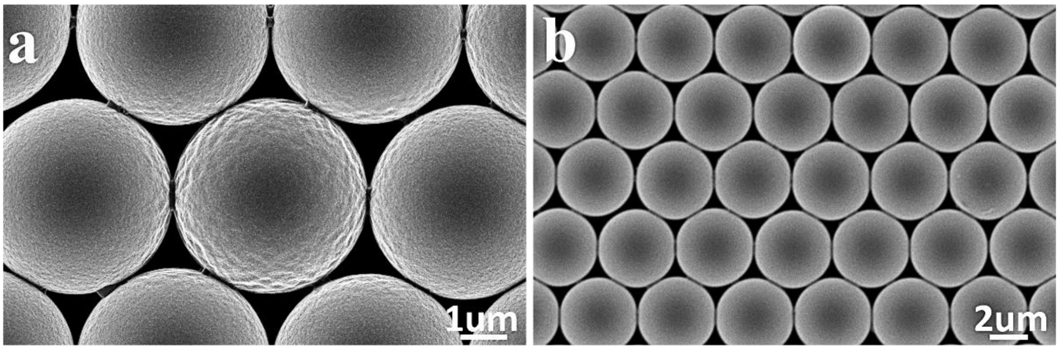

Figure S1. SEM images of the ZnO spherical shell coated the PS microspheres.

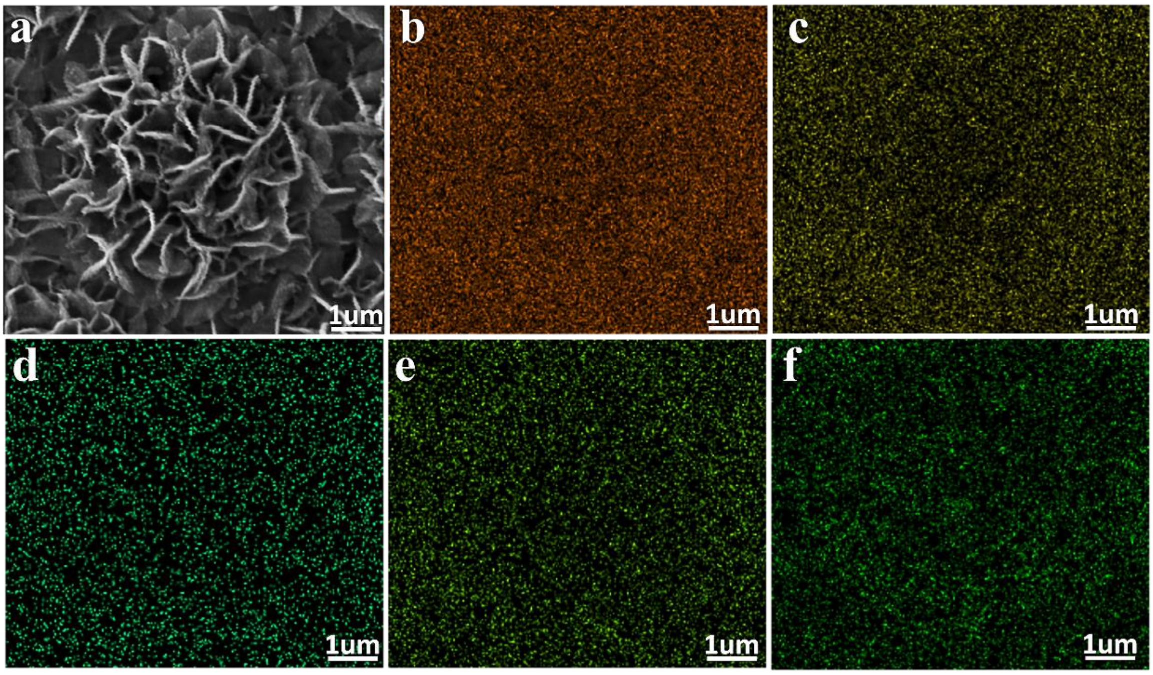

Figure S2. a) SEM images of $SnO_2/TiO_2/BiVO_4$ H-NSs@HMs arrays, Elemental mapping results b) Sn, c) Ti, d) Bi, e) V, f) O.

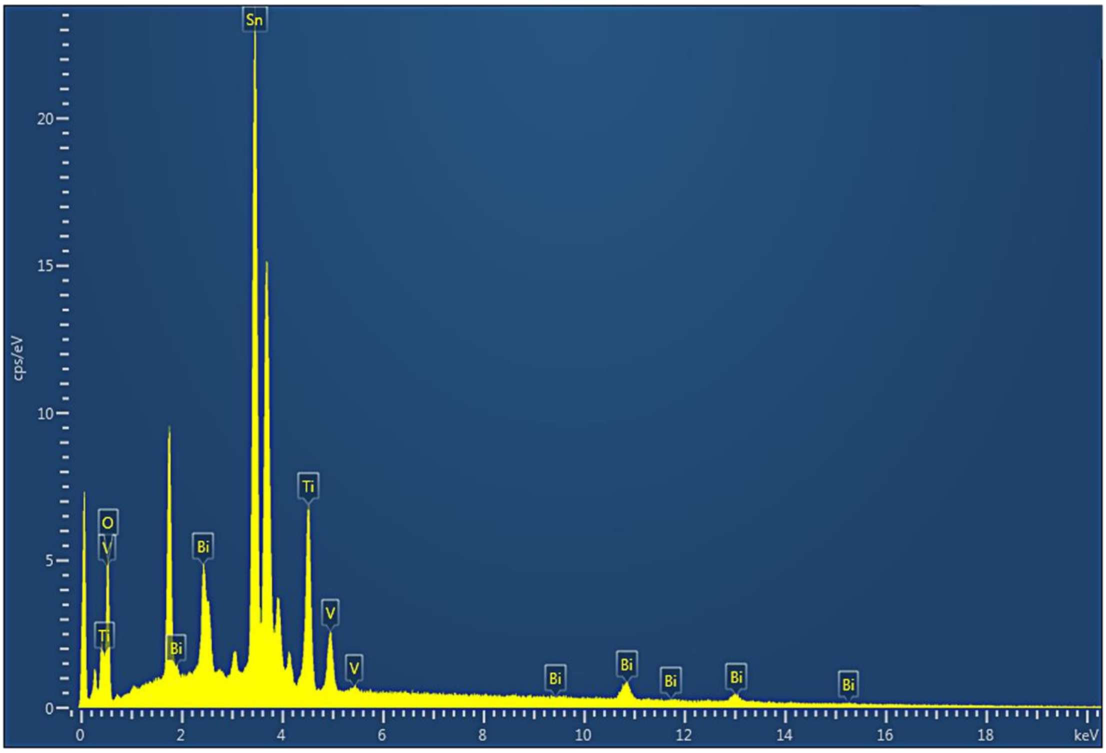

Figure S3. EDX spectra of the SnO_2/TiO_2/$BiVO_4$ H-NSs@HMs

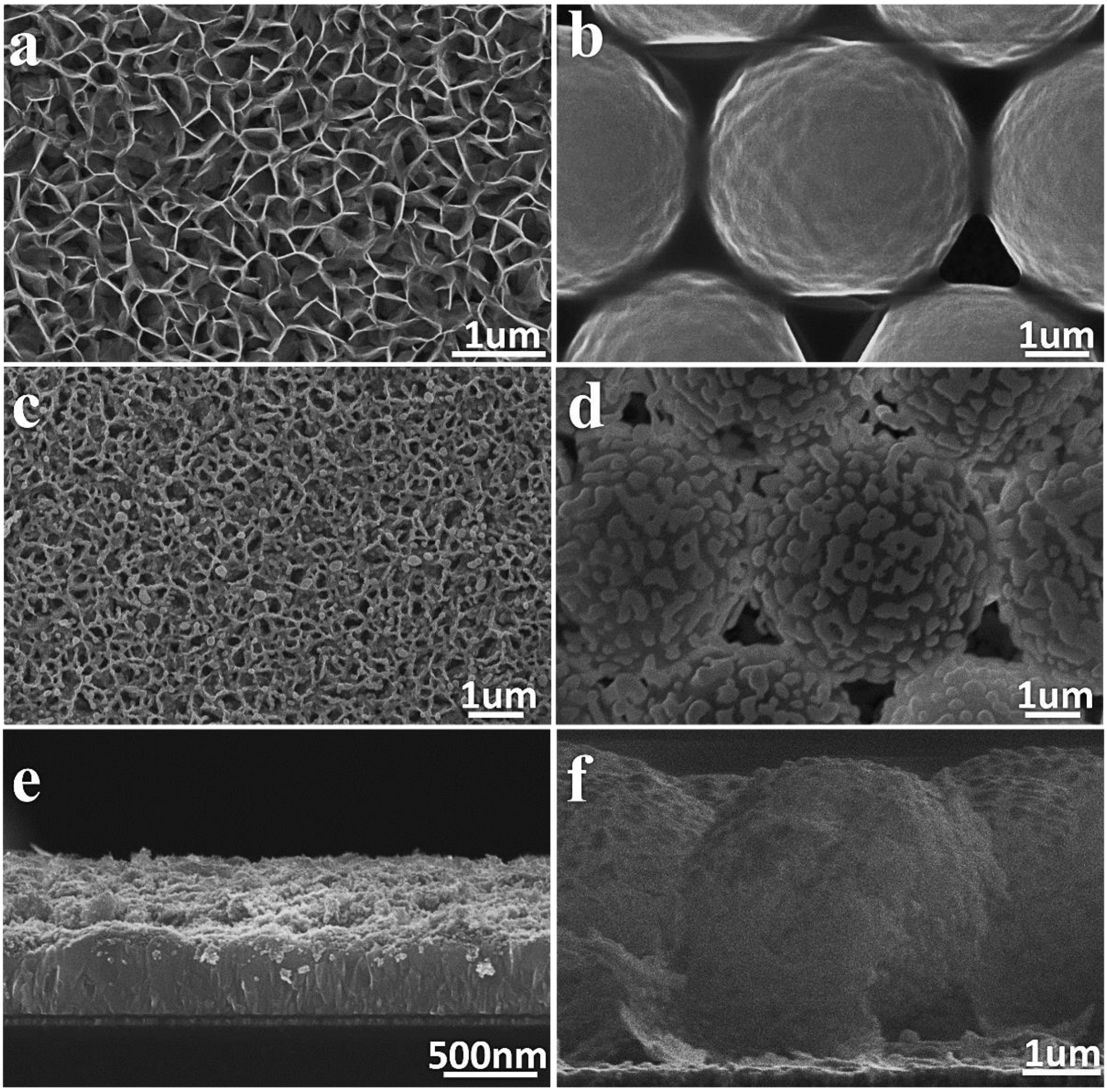

Figure S4. SEM images of a), c) $SnO_2/TiO_2/BiVO_4$ nanosheets, e) cross-section view; b) and d) $SnO_2/TiO_2/BiVO_4$ hollow microspheres arrays, f) cross-section view.

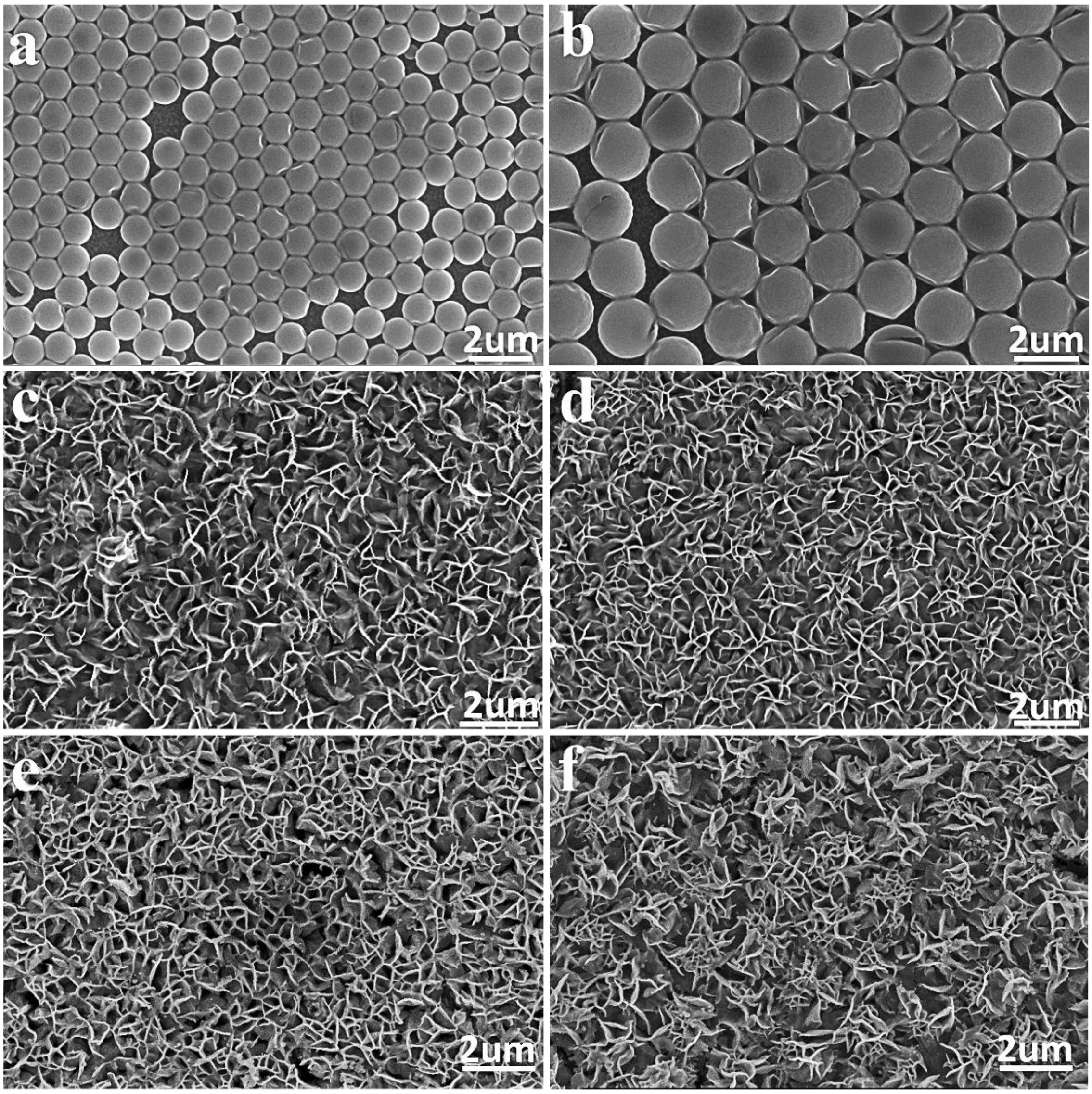

Figure S5. SEM images of a) SnO_2 hollow microsphere with diameters of 1μm; b) SnO_2 hollow microsphere with diameters of 1μm; c) SnO_2 H-NSs@HMs arrays with diameters of 1μm ; e) $SnO_2/TiO_2/BiVO_4$ H-NSs@HMs arrays diameters of 1μm; d) SnO_2 H-NSs@HMs arrays with diameters of 2 μm; f) $SnO_2/TiO_2/BiVO_4$ H-NSs@HMs arrays diameters of 2μm.

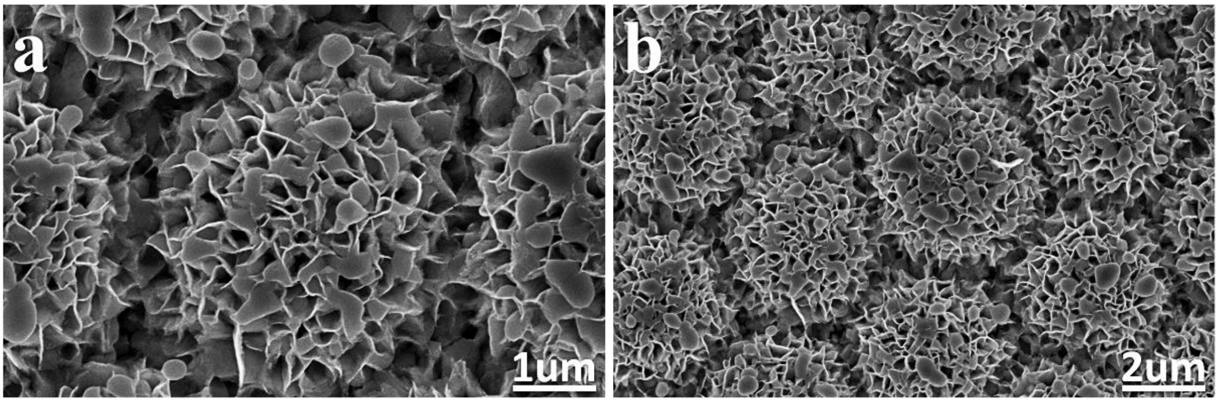

Figure S6. SEM images of $SnO_2/BiVO_4$ H-NSs@HMs arrays.

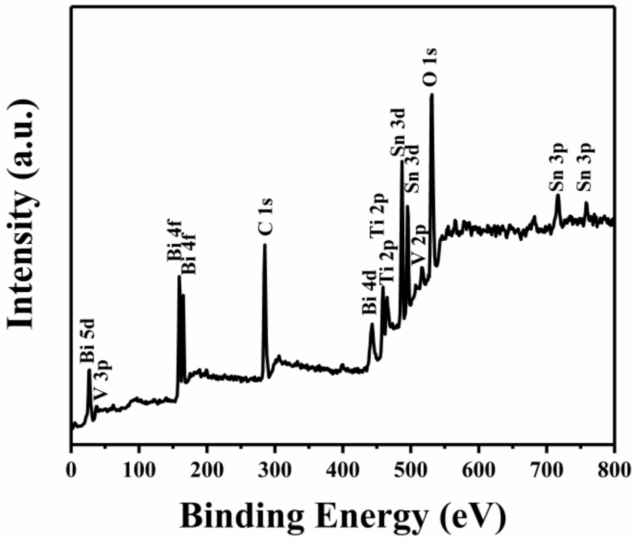

Figure S7. XPS full survey spectrum of $SnO_2/TiO_2/BiVO_4$ H-NSs@HMs.

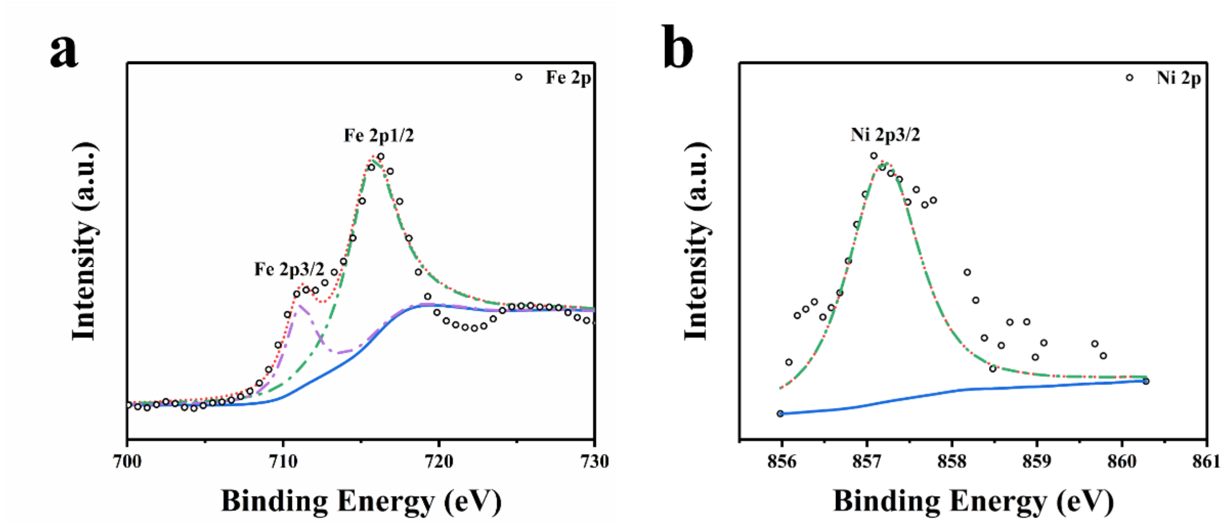

Figure S8. XPS core elemental spectra of FeOOH/NiOOH-modified $SnO_2/TiO_2/BiVO_4$ H-NSs@HMs: a) Fe 2p; b) Ni 2p.

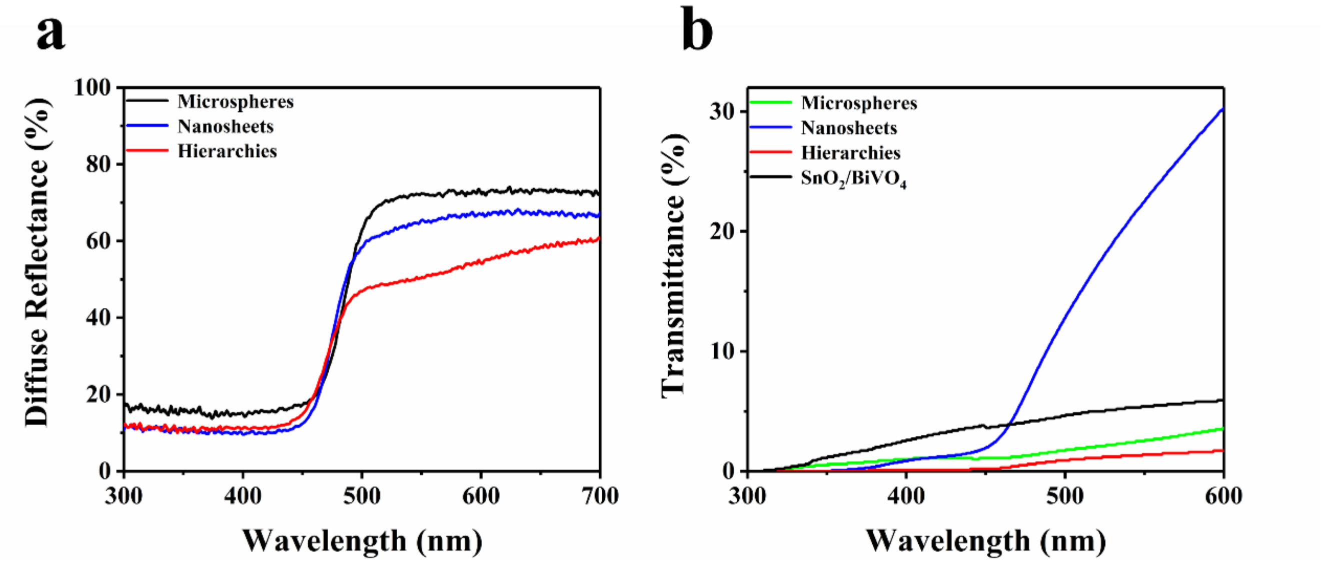

Figure S9. a) UV-vis diffuse reflectance spectra of SnO_2, SnO_2/TiO_2, $SnO_2/BiVO_4$, $SnO_2/TiO_2/BiVO_4$ H-NSs@HMs. b) Transmittance spectra of $SnO_2/TiO_2/BiVO_4$ microspheres, nanosheets, H-NSs @HMs and $SnO_2/BiVO_4$ H-Ns@HMs.

Table S1. The PEC performance comparison of $SnO_2/TiO_2/BiVO_4$ H-NSs/HMs other electrode inrecent reports.

**Photoanode**	**Electrolyte**	**Current densitidy (1.23V vs. RHE)**	**Refernce**
$SnO_2/TiO_2/BiVO_4$	0.5 M Na_2SO_4/ 0.1 M Na_2SO_3	5.04 mA/cm^2	This work
$NiOOH/BP/BiVO_4$	0.5 M KPi	4.48 mA/cm^2	[1]
$BiVO_4/SnO_2$ Nanorod	0.3 M Na_2SO_3	2.0 mA/cm^2	[2]
$SnO_2/BiVO_4$ Core−Shell	0.5 M Na_2SO_3	3.83 mA/cm^2	[3]
$BiVO_4$/FeOOH/NiOOH	1.0 KB	4.7 mA/cm^2	[4]
$BiVO_4$/Ag/rGO	0.5M Na_2SO_4/ 0.5 M Na_2SO_3	~4.2 mA/cm^2	[5]
Hierarchical $WO_3/BiVO_4$ Nanoporous Sphere	0.5 M KPi/ 0.5 M Na_2SO_3	5.5 mA/cm^2	[6]
Sb:$SnO_2/BiVO_4$ Danorod	0.5 M KPi/ 1 M Na_2SO_3	3.68 mA/cm^2	[7]
Ta:TiO_2|$BiVO_4$ Nanowire	0.5 M KPi	2.1 mA/cm^2	[8]
N_2-treated $BiVO_4$	0.5MKH_2PO_4/ 1 M Na_2SO_3	~5 mA/cm^2 (1.0V vs. RHE)	[9]
$BiVO_4$/Au Nanoparticles	MPi (pH=7)	~1.1 mA/cm^2	[10]
$BiVO_4$/FeOOH/NiOOH	0.5 M KPi/ 1 M Na_2SO_3	~4.7 mA/cm^2	[11]

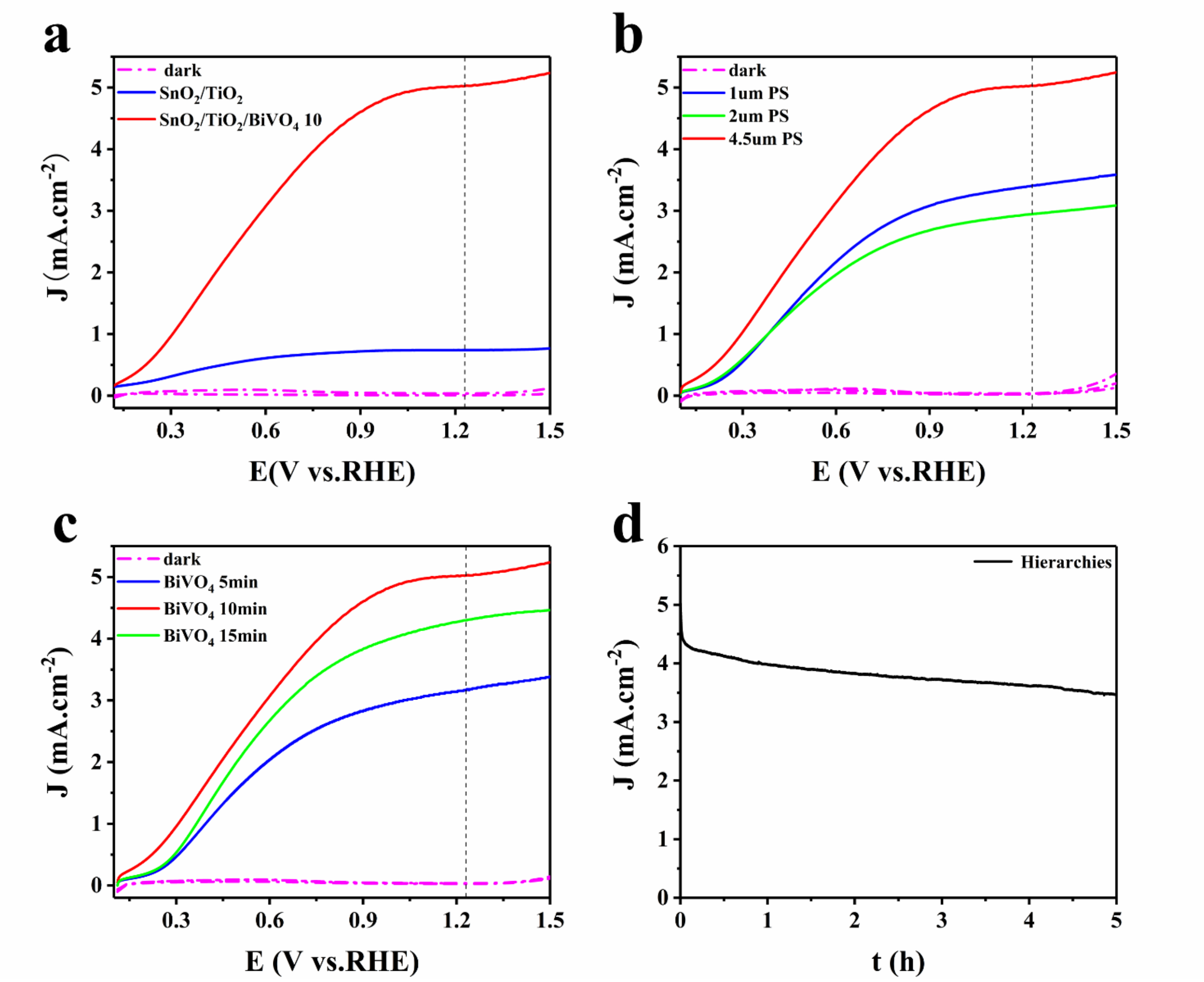

Figure S10. Linear sweep curve (LSV) of a) SnO_2/TiO_2 and $SnO_2/TiO_2/BiVO_4$ H-NSs@HMs. b) $SnO_2/TiO_2/BiVO_4$ H-NSs@HMs of 1 um, 2 um and 5 um PS. c) Comparison of different thicknesses (5 min, 10 min, 15 min) of $BiVO_4$ in the $SnO_2/TiO_2/BiVO_4$ H-NSs@HMs. d) Stability test of the $SnO_2/TiO_2/BiVO_4$ H-NSs@HMs.

S11 Calculation of the theoretical photocurrent in the photoanodes at the solar spectral irradiance [2].

The single photon energy is calculated by formula (1):

$$E(\lambda) = h \times C/\lambda \quad (1)$$

where $E(\lambda)$ is the photon energy, h is the planck's constant, C is the speed of light and λ is wavelength.

Solar photon flux is calculated by equation (2):

$$Flux(\lambda) = P(\lambda)/E(\lambda) \quad (2)$$

where $P(\lambda)$ is solar energy flux, a power function about λ. The theoretical maximum photocurrent density under standard Air Mass 1.5 normal spectral solar irradiance, J_{max}, is calculated by solar photon flux between 300 to 515 nm, shown in equation (3):

$$J_{max} = e \times \int_{300}^{515} Flux(\lambda) d\lambda \quad (3)$$

where e is electronic charge. In theory, J_{max} is consistently calculated to be 6.67 mA/cm^2 based our solar spectra.

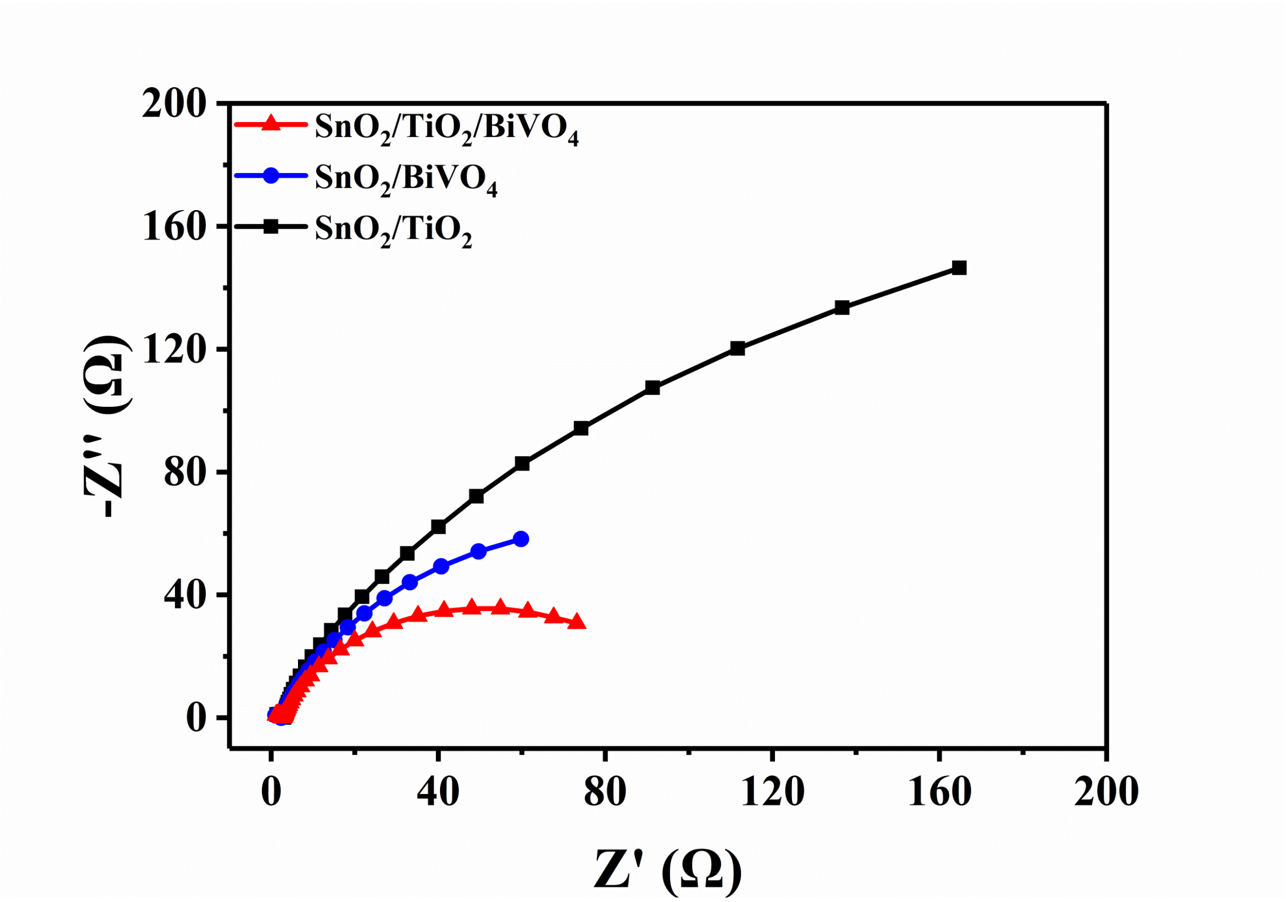

Figure S12. EIS spectra of (a) the $SnO_2/TiO_2/BiVO_4$, $SnO_2/BiVO_4$ and SnO_2/TiO_2 H-NSs@HMs.

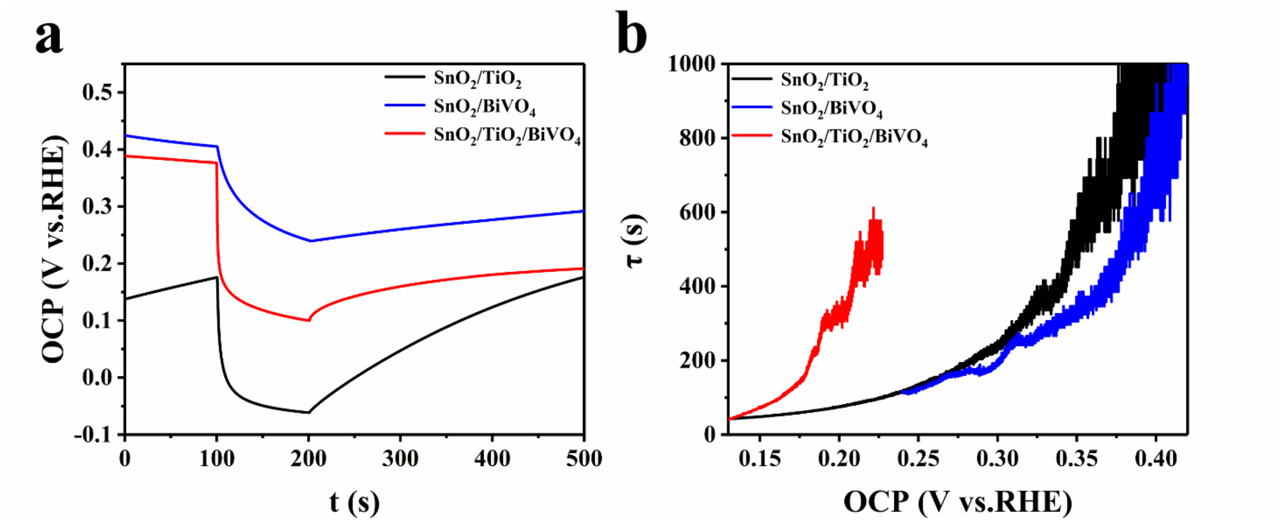

Figure S13. a), b) open circuit voltage decay (OCVD) and Electron lifetime of SnO_2/TiO_2 and SnO_2 $/BiVO_4$ H-NSs@HMs arrays.

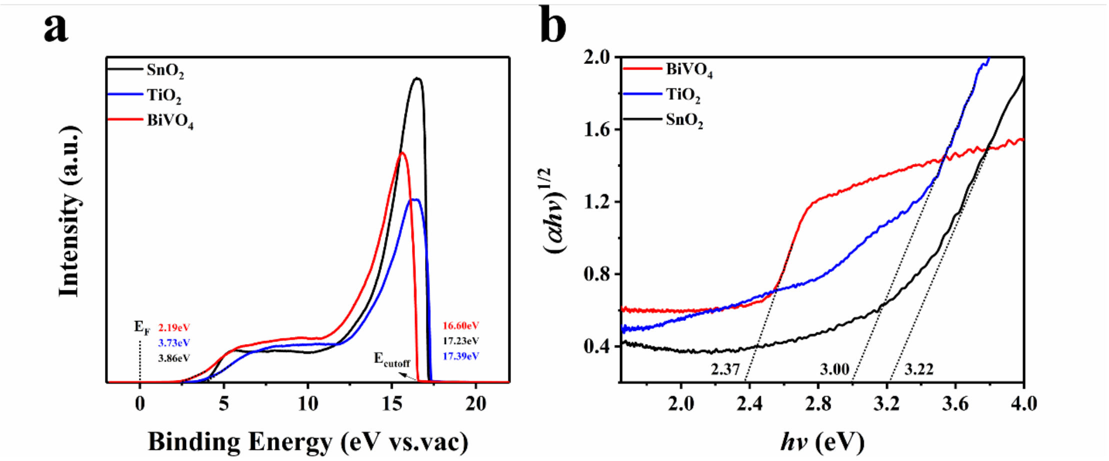

Figure S14 a) UPS spectra of SnO_2, TiO_2 and $BiVO_4$ with a fixed vacuum level. The Fermi level and cut off energy of the three semiconductors are indicated; b) Plots of transformed Kubelka–Munk function versus photon energy of SnO_2, TiO_2 and $BiVO_4$

Reference

[1] K. Zhang, B. J. Jin, C. Park, Y. Cho, X. F. Song, X. J. Shi, S. L. Zhang, W. Kim, H. B. Zeng, J. H. Park, *Nat. Commun.* **2019**, 10, 2001.

[2] S. Y. Chen, J. S. Yang, J. J. Wu, *ACS Appl. Energy Mater.* **2018**, 1, 2143.

[3] S. Bera, S. A. Lee, C. M. Kim, H. Khan, H. W. Jang, S. H. Kwon, *Chem. Mater.* **2018**, 30, 8501.

[4] D. K. Lee, K. S. Choi, *Nat. Energy* **2017**, 3, 53.

[5] S. S. Patil, M. G. Mali, M. A. Hassan, D. R. Patil, S. S. Kolekar, S. W. Ryu, *Sci. Rep.* **2017**, 7, 8404.

[6] Y. G. Zhou, L. Y. Zhang, L. H. Lin, B. R. Wygant, Y. Liu, Y. Zhu, Y. B. Zheng, C. B. Mullins, Y. Zhao, X. H. Zhang, G. H. Yu, *Nano Lett.* **2017**, 17, 8012.

[7] J. H. Kim, J. W. Jang, Y. H. Jo, F. F. Abdi, Y. H. Lee, R. V. Krol, J. S. Lee, *Nat. Commun.* **2016**, 7, 13380.

[8] L. Zhou, C. Q. Zhao, B. Giri, P. Allen, X. W. Xu, H. Joshi, Y. Y. Fan, L. V. Titova, P. M. Rao, *Nano. Lett.* **2016**, 16, 3463.

[9] J. Resasco, H. Zhang, N. Kornienko, N. Becknell, H. Lee, J. H. Guo, A. L. Briseno, P. D. Yang, *ACS Cent. Sci.* **2016**, 2, 80.

[10] T. W. Kim, Y. Ping, G. A. Galli, K. S. Choi, *Nat. Commun.* **2015**, 6, 8769.

[11] T. W. Kim, K. S. Choi, *Science* **2014**, 343, 6174, 990.
